# Perforation of Jejunum as a Rare Complication of Pericardiocentesis: A Case Report

**DOI:** 10.1002/ccr3.72348

**Published:** 2026-05-10

**Authors:** Fatemeh Bahrami, Maryam Mehrpooya, Seyed Mohsen Ahmadi

**Affiliations:** ^1^ Department of Cardiology, Imam Khomeini Hospital Complex Tehran University of Medical Science Tehran Iran; ^2^ Interventional Cardiology, Department of Cardiology, Imam Khomeini Hospital Complex Tehran University of Medical Science Tehran Iran; ^3^ Division of Colorectal Surgery, Department of Surgery, Imam Khomeini Hospital Complex Tehran University of Medical Science Tehran Iran

**Keywords:** bowel perforation, case report, laparotomy, pericardiocentesis, tamponade

## Abstract

The safety of pericardiocentesis as a therapy for cardiac tamponade has improved since the advent of echocardiography‐guided pericardiocentesis. The most serious complications include injury of the cardiac chambers, laceration of the coronary arteries or intercostal vessels, puncture of the abdominal viscera or peritoneal cavity, pneumothorax, pneumopericardium, ventricular arrhythmias, pericardial decompression syndrome, myocardial and coronary puncture. Iatrogenic intestinal injury during an attempt for pericardiocentesis is very rare; in addition, the diagnosis of this complication may be very challenging. We present a rare case of perforation of the jejunum caused by pericardiocentesis, which was promptly diagnosed and managed emergently.

## Key Clinical Massage

The purpose of sharing this case is to explain the possibility of rare complications of pericardiocentesis, which shows the necessity of using all diagnostic methods and precision in guiding the needle, even in routine and life‐saving procedures, including pericardiocentesis.

## 1 Introduction

Pericardiocentesis is an invasive procedure used for the treatment of pericardial tamponade, and also obtained pericardial fluid used for diagnostic purposes [1]. In the past, the most indications of pericardiocentesis included tamponade due to tuberculous pericarditis, uremia, and malignant pericardial effusions. But recently incidence of iatrogenic pericardial effusions is becoming more frequent because the number of catheter‐based interventional cardiac procedures has increased in the last decades 1 [2]. The subxiphoid approach for insertion of a needle is more common. In this approach the needle insertion site is between the Xyphosternal angle and the left costal margin [3]. Guidance of echocardiography or fluoroscopy generally results in more than95% success rates and decreases the complication rate to 1%–2% [1]. Major complications include ventricular perforation, infection, pneumothorax, ventricular arrhythmias, cardiac arrest, pericardial thrombus, and pulmonary edema. Minor complications include transient arrhythmias, small pneumothorax, and pericardial catheter (pigtail) occlusion [4, 5]. We reported a rare complication of pericardiocentesis by subxiphoid approach. Identification of this complication, in addition to timely management, is crucial for saving a patient's life.

## 2 Case Report

### 2.1 History of Presentation

A 64‐year‐old man with a history of malabsorption admitted with severe cachexia. He had recent worsening of dyspnea in New York Heart Association (NYHA) functional class III. He had also recent admission 10 days ago for more evaluation of malabsorption symptoms and had undergone laparoscopic investigation, but it wasn't diagnostic. He was bedridden from 1 month ago. He had orthopnea but denied paroxysmal nocturnal dyspnea. Vital signs included a heart rate of 110 beats/min, blood pressure of 85/65 mmHg, and an oxygen saturation of 92% on room air. Cardiac examination revealed distant heart sounds with difficult audible S1 and S2 and no obvious friction rub, gallop, or murmur. pulsus paradoxus was absent. Abdomen was distended and lower limb edema was present. The rest of the examination was unremarkable.

### 2.2 Past Medical History

He had a history of malabsorption since 6 years ago and significant cachexia since 2 months ago.

### 2.3 Differential Diagnosis

Initial differential diagnoses included pneumonia, heart failure, recurrent viral or autoimmune pericarditis, and cardiac tamponade.

### 2.4 Investigations

Results of the complete blood count showed anemia. Liver enzymes were elevated. Thyroid studies and cardiac biomarkers were normal. ESR and CRP were elevated.

Electrocardiogram (ECG) and transthoracic echocardiogram (TTE) were done for evaluation of dyspnea. An electrocardiogram showed sinus tachycardia, normal‐axis, and low voltage criteria in limb leads (Figure 1).

**FIGURE 1:**
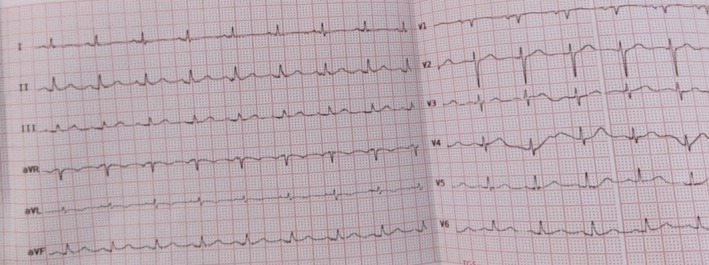
Electrocardiogram showed sinus tachycardia, normal‐axis and low voltage criteria in limb leads.

In TTE, left ventricle function (LV) was preserved (EF = 50%–55%) and severe circumferential pericardial effusion was detected (30 mm posterior to LV, 25 mm anterior to RV) with significant mitral and tricuspid inflow respiratory variation also right atrium (RA) and right ventricular (RV) collapse during whole diastolic period. Dilated inferior vena cava (30 mm) without respiratory collapse was also seen. (Figures 2 and 3).

**FIGURE 2:**
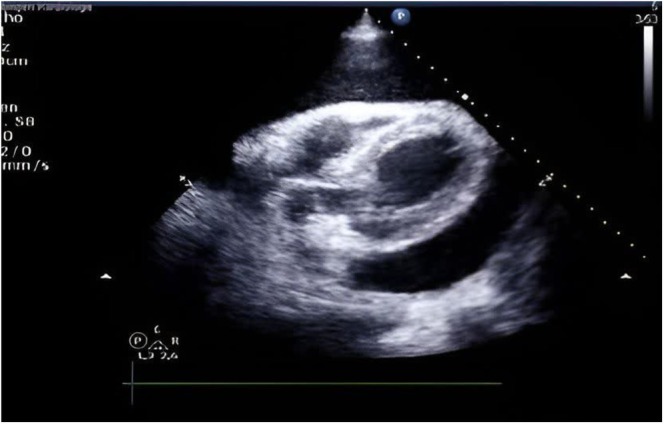
Severe pericardial effusion was detected with in TTE.

**FIGURE 3:**
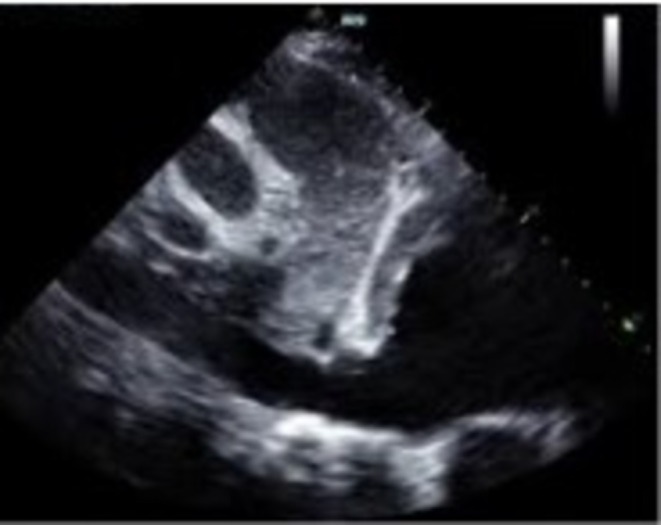
Dilated inferior vena cava without respiratory collapse in TTE.

### 2.5 Management

The patient continued to have dyspnea and became tachycardic. Given the echocardiographic findings, the patient's symptoms were attributed to impending cardiac tamponade, and a decision was made to proceed with pericardiocentesis. He was taken to the cardiac catheterization laboratory. Echocardiography wasn't helpful in the correct positioning of needle; therefore the needle was inserted via subxiphoid approach under fluoroscopic guidance (seemingly in the right usual place) with dye swirling (Video 1), then the wire was placed, and the needle was removed, and the sheet was inserted, then the pigtail was inserted through the wire into the cavity that appeared to be the pericardium. In spite of the weird course of wire pigtail was advanced (Video 2) but the drainage fluid was dark green, having very bad smell, and the patient had abdominal pain. The pigtail was removed and the fluoroscopy movies were reviewed again. At that time, we made sure that the injection into the needle, course of wire, sheet and pigtail insertion sites was abnormal. In fact, the sheet was implanted in the intestine, unfortunately.

**VIDEO 1:** Needle was inserted via subxiphoid approach under fluoroscopic guidance (seemingly in the right usual place) with dye swirling. Video content can be viewed at https://onlinelibrary.wiley.com/doi/10.1002/ccr3.72348.

**VIDEO 2:** Abnormal course of wire in the intestine. Video content can be viewed at https://onlinelibrary.wiley.com/doi/10.1002/ccr3.72348.

The patient was hemodynamically stable. Regarding surgery consultation, the sheath was removed and the patient was transferred for an abdominal graph and abdominopelvic (computed tomography) CT. Bowel perforation (jejunum) was confirmed by abdominopelvic CT (Figures 4 and 5) and the patient was transferred for open surgery.

**FIGURE 4:**
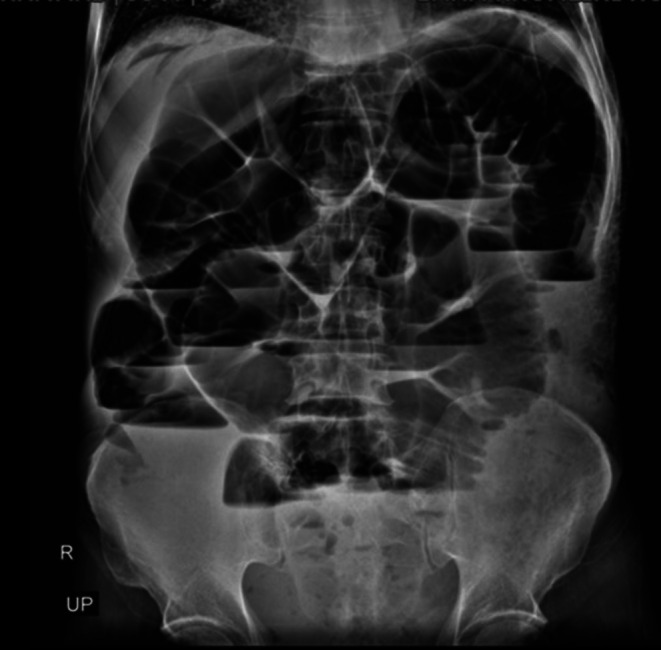
Abdominal upright graph showed dilated intestine, and the presence of air below the diaphragm was compatible with bowel perforation.

**FIGURE 5:**
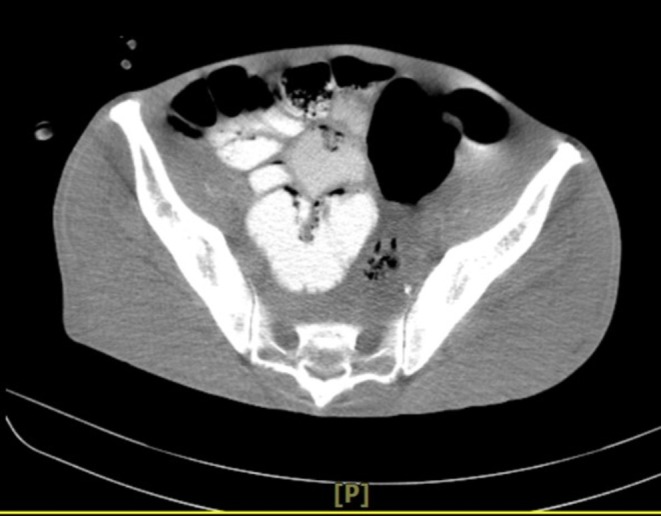
Abdominopelvic CT showed fluid in abdominal space and dilated intestine compatible with bowel perforation (jejunum).

Laparotomy showed a very distended intestinal loop and jejunum repaired surgically (Figure 6). Pericardiocentesis was performed under the guidance of echocardiography with direct assistance and supervision of the surgeon, and 1500 cc of dark yellow fluid was drained. Due to the disappearance of findings of tamponade in echocardiography and prevention of infection, a pigtail was not inserted. In spite of that, exploratory laparotomy was part of the diagnostic procedures for intestinal obstruction, but there wasn't physical obstruction in the intestines, and mostly ileus was considered. His blood pressure was increased to 110/76 mmHg, heart rate reached 78 beat/min, and oxygen saturation became 93%. After 1 day stay in the intensive care unit and initial stabilization, he was transferred to the gastroenterology ward. Patient's symptoms were diminished in 2 days. Some care was done for his malabsorption syndrome which was compatible with celiac disease. In 2 month follow‐up, his clinical course was uneventful.

**FIGURE 6:**
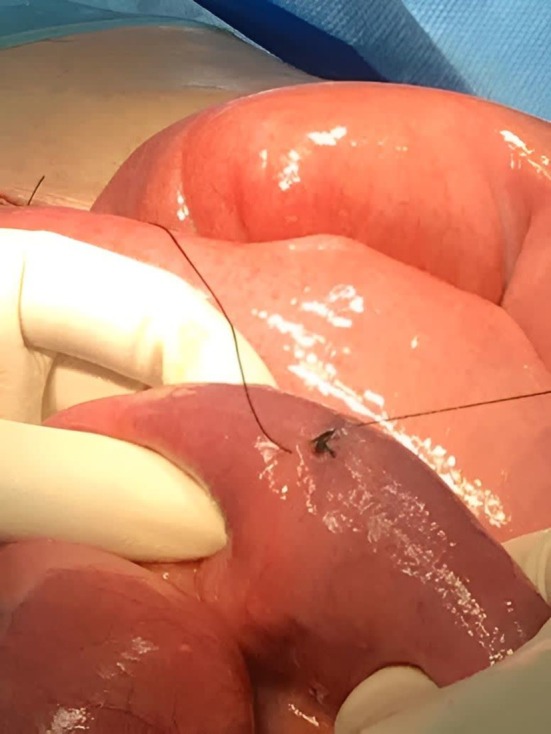
Laparotomy showed very distended intestinal loop and jejunum repaired surgically.

Finally, besides early diagnosis and management, a multidisciplinary approach, including an interventional cardiologist and gastrointestinal surgeon was an important team collaboration for preserving the patient ‘life and subsequent problems.

## 3 Discussion

We reported a case of perforation of the jejunum due to pericardiocentesis. Bowel perforation is a rare complication of pericardiocentesis. The procedure of pericardiocentesis was performed blindly until 1840. “Blind approach” was associated with increased morbidity and mortality rates, but with the improvement of imaging guidance, the rates of major and minor complications have decreased remarkably [2, 4].

Major complications include ventricular perforation, infection, pneumothorax, ventricular arrhythmias, cardiac arrest, pericardial thrombus, and pulmonary edema. Minor complications include transient arrhythmias, small pneumothorax, and pericardial catheter (pigtail) occlusion [4, 5, 6]. Other rare complications reported as a case report include hem peritonitis and peritonitis due to left inferior phrenic artery or liver, spleen, and diaphragm injury [1].

In a case report by Albalat et al. [7], hemoperitoneum due to left inferior phrenic artery injury during pericardiocentesis was described, which was managed surgically to stop the bleeding.

Perforation of the Jejunum due to pericardiocentesis and discharge of intestinal contents from the inserted catheter haven't been reported yet. Regarding our vast investigation, there have been three other cases of bowel perforation due to pericardiocentesis. Duvernoy et al. reported two cases of bowel perforation after pericardiocentesis, but they were managed conservatively without surgical intervention [8].

Peritonitis and pneumoperitoneum after pericardiocentesis in the setting of interposition of the colon between liver and diaphragm (so‐called Chilaiditi syndrome) has also been described. In the mentioned case report, the patient's symptoms were diminished and signs of peritoneal infection resolved after 48 h of conservative therapy, without the need for invasive management [9].

Bowel perforation secondary to pericardiocentesis is rare, and so there are no evidence‐based consensus recommendations for surgical or nonsurgical management. Most authors recommend operative therapy in cases with large perforations and signs of peritonitis, whereas smaller perforations may often be treated nonsurgically; however, mostly previous clinical experience with perforation was secondary to polypectomy or colonoscopy, not as a result of pericardiocentesis [9]. Use of both echocardiography and fluoroscopy guidance for this procedure is necessary to minimize any potential complications, some of which could be lethal.

In our case, in spite of the usual subxiphoid approach for pericardiocentesis and also dye injection under fluoroscopy guidance, the severely distended and elevated intestines predisposed it to perforation, and surgical management was chosen regarding multidisciplinary decision making.

## 4 Conclusion

Imaging‐guided pericardiocentesis, especially the echo‐guided procedure, has significantly improved the safety and feasibility of pericardiocentesis and helps to choose the best anatomical approach. In addition, in any pericardiocentesis, it is necessary to pay attention to possible complications, even rare ones.

## Author Contributions

**Fatemeh Bahrami:** investigation, writing – original draft, writing – review and editing. **Maryam Mehrpooya:** conceptualization, data curation, methodology, project administration, writing – original draft, writing – review and editing. **Seyed Mohsen Ahmadi:** investigation, supervision.

## Funding

The authors have nothing to report.

## Consent

Written informed consent was obtained from the patient to publish this case report in accordance with the journal's patient consent policy.

## Conflicts of Interest

The authors declare no conflicts of interest.

## Data Availability

The data of this study are available for further assessment.
